# Genomic continuity of Argentinean Mennonites

**DOI:** 10.1038/srep36392

**Published:** 2016-11-08

**Authors:** Jacobo Pardo-Seco, Cintia Llull, Gabriela Berardi, Andrea Gómez, Fernando Andreatta, Federico Martinón-Torres, Ulises Toscanini, Antonio Salas

**Affiliations:** 1 Unidade de Xenética, Departamento de Anatomía Patolóxica e Ciencias Forenses, Instituto de Ciencias Forenses, Facultade de Medicina, Universidade de Santiago de Compostela, and GenPop Research Group, Instituto de Investigaciones Sanitarias (IDIS), Hospital Clínico Universitario de Santiago, Galicia, Spain; 2 Grupo de Investigación en Genética, Vacunas, Infecciones y Pediatría (GENVIP), Hospital Clínico Universitario and Universidade de Santiago de Compostela (USC), Galicia, Spain; 3 PRICAI-Fundación Favaloro, Buenos Aires, Argentina; 4 Hospital Dr Manuel Freire, Guatrache, La Pampa, Argentina; 5 Infectious Diseases and Vaccines Unit, Department of Pediatrics, Hospital Clínico Universitario de Santiago, Santiago de Compostela, Galicia, Spain

## Abstract

Mennonites are Anabaptist communities that originated in Central Europe about 500 years ago. They initially migrated to different European countries, and in the early 18^th^ century they established their first communities in North America, from where they moved to other American regions. We aimed to analyze an Argentinean Mennonite congregation from a genome-wide perspective by way of investigating >580.000 autosomal SNPs. Several analyses show that Argentinean Mennonites have European ancestry without signatures of admixture with other non-European American populations. Among the worldwide datasets used for population comparison, the CEU, which is the best-subrogated Central European population existing in The 1000 Genome Project, is the dataset showing the closest genome affinity to the Mennonites. When compared to other European population samples, the Mennonites show higher inbreeding coefficient values. Argentinean Mennonites show signatures of genetic continuity with no evidence of admixture with Americans of Native American or sub-Saharan African ancestry. Their genome indicates the existence of an increased endogamy compared to other Europeans most likely mirroring their lifestyle that involve small communities and historical consanguineous marriages.

Anabaptists represent a Christian movement that traces its origins to the 16^th^ century, at the time of the Radical Reformation in Europe. It differentiates from other movements in their assertion of the necessity of adult baptism, in contrast to the infant baptism practiced by the Roman Catholic Church^1^. There are several Anabaptists communities, as for instance, the Amish, Hutterites, and Mennonites; being the Amish the largest one. The Mennonites separated from the Amish already in the late 1600’s, and they slightly diverged from Amish in regards to their costumes and lifestyle (http://jamesportmissouri.org/index.html, refs 2 and 3). Their denomination comes from the Anabaptist religious leader Menno Simons (1596–1561).

Initial migrations of Mennonites to America originated in the Netherlands and West Germany (Krefeld) and disembarked in New York and Germantown in the 17^th^ century. Prussian Mennonites mainly settled in the Unites States, while the Chostitza (present-day Ukraine) migrants largely moved to the Manitoba province in Canada. Starting in the early 1920s, some colonists migrated from Canada to the provinces of Chihuahua and Durango in Mexico, and to a lesser extent to different destinations in South America, namely Paraguay, Bolivia, Uruguay and Brazil.

Nowadays, there are about 1.8 million Mennonites worldwide (http://gameo.org; 2012), and about 80.000 live in South America, most of them in Paraguay, Bolivia and Brazil. Small communities were also established in Argentina and Uruguay very recently.

The Argentinean Mennonite Colony ‘La Nueva Esperanza’ was established in the mid 1980s in the Department of Guatraché, in La Pampa province, where other immigrant communities of non-Mennonite origin also exist nowadays^2^,^4^. The first settlers arrived in 1985, when they initially acquired the lands that would later be structured and subdivided according to their socio-political rules. The founding members of ‘La Nueva Esperanza’ originated from the Mexican and Bolivian communities, and belong to the Anabaptist branch known as *Ältkolonier Reinlaender Mennoniten Gemeinde* (ÄRMG) or ‘The Community of Mennonites from the Old Colony of Reinland’, who initially reached these latitudes in search of new horizons to expand their population where their religious, educational and socio-cultural traditions could be respected, also in a legal state frame^2^,^3^,^5^,^6^,^7^. Historical records indicate that pioneers of the Argentinean Mennonite community came from Chostitza and ultimately Central Europe, pointing to the Netherlands as the core of the original Mennonite diaspora^3^,^8^.

Apart from the early studies on classical markers (e.g. refs 5 and 6), only a few molecular genetic studies have been carried out on Mennonite communities^3^,^9^,^10^; a few other studies have focused on other non-Mennonite Anabaptist communities^11^. Thus, two very early studies carried out by Jaworsky *et al*.^12^,^13^ analyzed genetic conditions among Canadian Mennonites in regards to the old colony (Chortitza) Mennonites; the authors focused on their singular epidemiological characteristics as well as molecular genetic features related to common and rare inherited disease. The more recent study by Demarchi *et al*.^14^ analyzed apoliproproteins and LPL variation in Mennonites from Kansas and Nebraska, the study indicating important frequency differences with respect to other Europeans. The analysis carried out by Demarchi and colleagues could not however identify the causes for such differences. Strauss and Puffenberger^15^ reviewed medical genetic conditions of Mennonites from Pennsylvania, highlighting the need of these medical studies for disease prevention and reduction of medical costs in these communities. The two most recent articles carried out on Mennonites focused on the analysis of uniparental markers in a population genetic context. Thus, Melton *et al*.^10^ sequenced segments of the mitochondrial DNA (mtDNA) control region of Mennonite communities from the USA; the authors reported the non-admixture nature of these communities. Males from the congregation of ‘La Nueva Esperanza’ were recently analyzed for a selection of Y-chromosome short tandem repeats (YSTRs); ref. 9. This study revealed that the Y-chromosomes of Mennonites from ‘La Nueva Esperanza’ are genetically related to populations living in Central Europe, with the Netherlands being the population that best represents the genetic background of this community. The sample from ‘La Nueva Esperanza’ exhibited lower variability when compared to variation observed in neighboring populations, which was attributed to a higher level of endogamy. Besides, analyses of male lineages in this population did not show signals of admixture with Native American, African, or other local groups. However, it is important to note that Y-chromosome analysis may fail to detect admixture when the inbreeding with local people is low and/or when the sample size is limited^9^.

The aim of the present study was to carry out a genome-wide study of the Mennonite community of ‘La Nueva Esperanza’. Genome-wide studies offer several advantages with regards to uniparental markers. For instance, single genomes can be informative concerning historical admixture and demography (e.g. refs 16 and 17). It was expected that results of this analysis would shed light on the genetic background of Mennonites and be of interest from socio-anthropological, medical, and forensic genetic perspectives.

## Results

### Identity-by-descent, identity-by-state, and multidimensional scaling analysis

**Figure 1:**
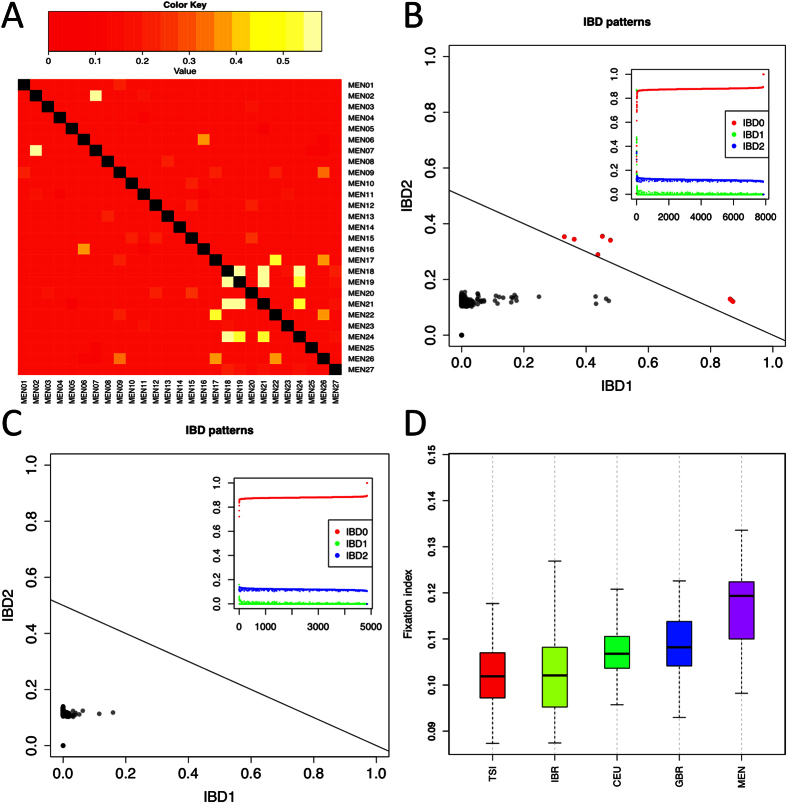
(**A**) Cryptic relatedness of Mennonites inferred from pi_hat values, which were computed as p(IBD = 2) + 0.5 × p(IBD = 1) (ref. 23); (**B**) IBD values in Argentinean Mennonites; (**C**) IBD values in the CEU reference population (from 1000G); and (**C**) F-inbreeding coefficient boxplots for Mennonites and European population sets.

Analyses of identity-by-descent (IBD) values were carried out in order to detect close relationships between individuals that could not be detected based on the genealogical information provided by the donors. This analysis allowed the identification of a few cryptic relationships (Fig. 1A). Figure 1B summarizes IBD values in Mennonites, while Fig. 1C uses the CEU population for comparison. A total of five individuals showing IBD values >0.5 were eliminated to prevent interference with other analyses.

F-inbreeding coefficients (fixation indexes) were obtained for Mennonites and other reference European samples from the 1000 Genome Project (hereafter 1000G). Mennonites show the highest fixation index when compared to Europeans (Fig. 1B), and this difference is statistically significant (Mann-Whitney test, *P*-value < 0.001).

**Figure 2:**
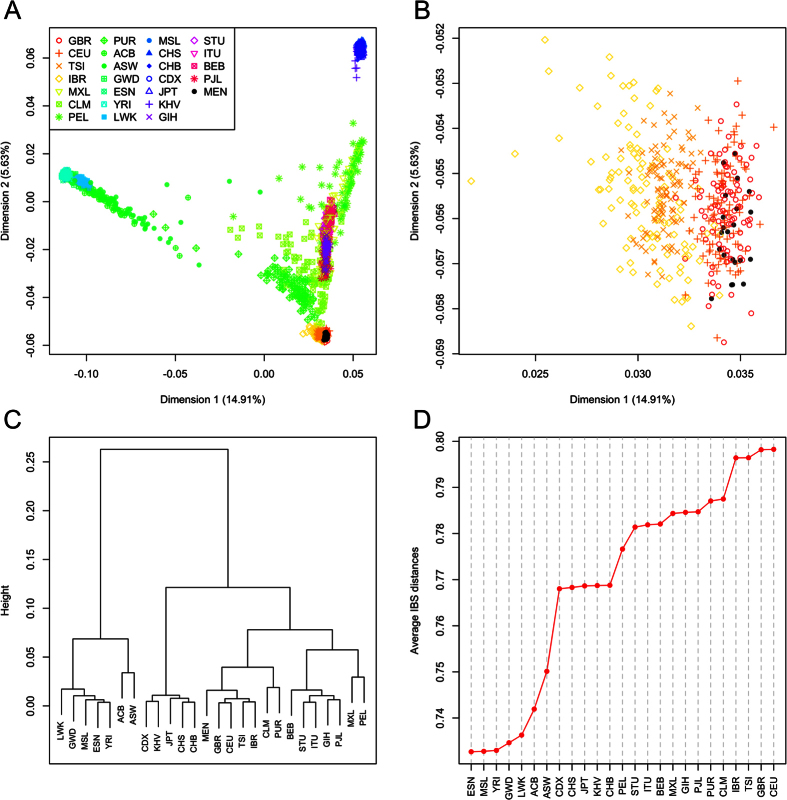
(**A**) MDS plot of Mennonite genetic variation in the context of worldwide population samples. (**B**) Detail of the European pole in the MDS plot of (**A**). (**C**) Dendogram based on IBS values. (**D**) Average IBS values in the different popul × ations used for comparison with the Mennonites.

The Multidimensional-scaling (MDS) plot based on identity-by-state (IBS) values (Fig. 2A) displays three main clusters located at the vertices of a triangle, and representing the three main continental groups, Europe, Africa and Asia. While Dimension 1 (accounting for ~15% of the variation) separates African profiles from European and Asian ones, Dimension 2 (~6%) separates Asian from European and African profiles. A number of samples from different population groups fall in between these three main clusters, reflecting the existence of two or three-side admixture. Mennonite variation falls perfectly within the European cluster, with no evidence of admixture with other continental groups. Within Europe, Mennonites cluster together with the CEU and the GBR samples (Fig. 2B).

The dendogram in Fig. 2C identifies the Mennonites within the European cluster, with the CEU being the most closely related population set. Average IBS distances (Fig. 2D) between Mennonites and reference populations indicate that Mennonites are closely related to other Europeans from 1000G. In particular, the CEU is the population with closest genetic affinity with the Mennonites.

*F*_*ST*_ were also computed between Mennonites and different datasets. In good agreement with the analysis of IBS, the data indicate that the Mennonites are closely related to the European datasets. The highest *F*_*ST*_ were found to be with Africans and Asians (Figure S1). A MDS plot based on *F*_*ST*_ values reasserts this observation, while a dendogram indicates that the Mennonites are located in the European cluster, in a separate branch to the rest of the other European datasets used (Figure S1).

Additional analysis carried out using other European population sets form Behar *et al*.^18^ and Human Genome Diversity Project^19^ (data not shown) also indicate that the CEU is the most closely related population to the Mennonites.

### Admixture analysis of Mennonite genomic profiles

**Figure 3:**
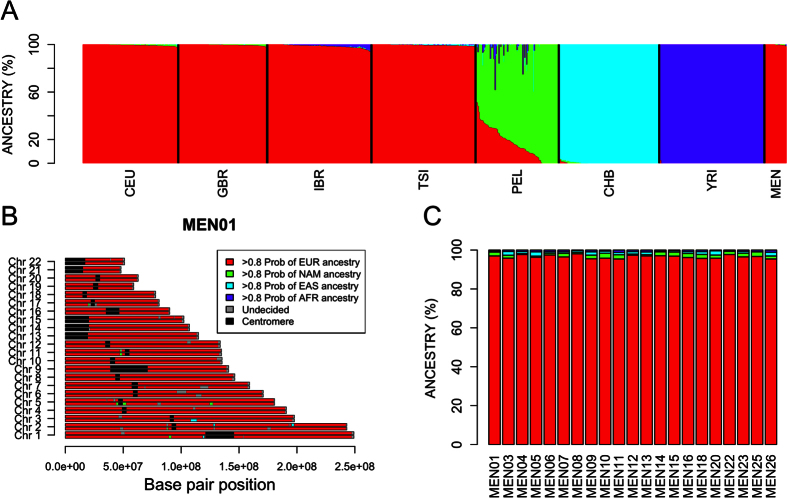
(**A**) Analysis of admixture of Mennonites using ADMIXTURE. The bar-plot represents individual ancestries in reference populations and Mennonites. The analysis was carried out using the unsupervised clustering algorithm implemented in ADMIXTURE and considering the run with the lowest cross validation value. (**B**) Genomic (chromosome) ancestry mosaic for one Mennonite (#MEN01) using PCAdmix (reference populations: CEU, YRI, CHB, PEL). (**C**) Admixture proportions of Mennonites using PCAdmix (reference populations: CEU, YRI, CHB, PEL).

Admixture analysis was carried out using a selection of 1000G population sets that represent the main continental ancestries. With the selected populations, *K* = 4 yielded the analysis with the lowest cross validation value; this analysis classifies profiles into four main groups that coincide with Europeans, Native Americans (represented here by Peruvians), East Asians, and Sub-Saharan Africans (Fig. 3A). This analysis allocates most of the Mennonite genomic variation (99.7% on average) to European ancestry.

The results of PCAdmix show virtually the same results (Fig. 3B,C), with ~97% of the ancestry allocated to the European cluster.

### *D*-statistics

**Figure 4:**
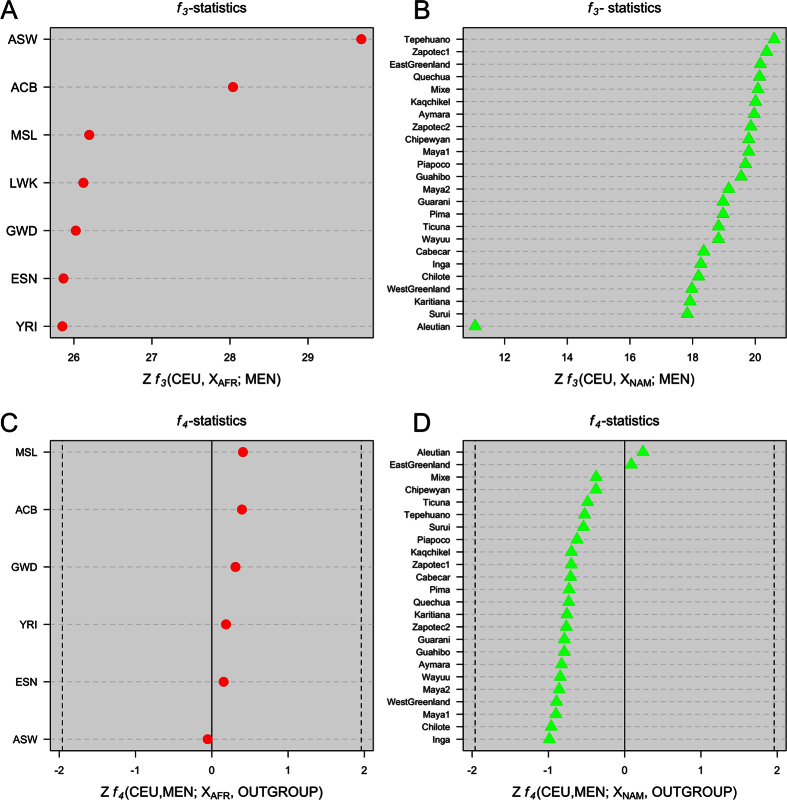
*f*_*3*_-statistics (**A,B**) and *f*_*4*_-statistics (**C,D**) of Mennonites *versus* different sub-Saharan (**A,C**) and Native American (**B,D**) populations.

The *f*_*3*_-statistic test determines whether the relationships between three populations can be explained with or without admixture, while the *f*_*4*_-statistic adds additional information about the direction of the gene flow. We carried out these analyses in order to explore if the Mennonite profiles could be explained by recent admixture between European variations and African and/or Native American ones. Both *f*_*3*_-statistics and *f*_*4*_-statistics showed no evidence of admixture (Fig. 4).

## Discussion and Conclusions

The present study represents the first attempt aimed at exploring genome-wide SNP variation of Mennonites. Previous efforts were carried out on uniparental markers, using Y-STRs and mtDNA control region variation^3^,^9^,^10^. Uniparental markers have however some limitations for the interpretation of historical admixture when explored individually.

The historical routes followed by Mennonite communities once they arrived to America from European countries exposed them to population scenarios that favored conditions for admixture with locals of African and Native American ancestry. Our data indicate however that the variation observed in Mennonites is virtually of 100% European ancestry. The Native American ancestry identified by ADMIXTURE and PCAdmix, apart from being very low, is most likely due to the presence of European component in the Native American populations used as references (see barplot of PEL in Fig. 3). In fact, admixture and PCAdmix analysis using other reference Native American populations, e.g. Aymara and Quechua samples from Reich *et al*.^20^, corroborate a virtually 0% Native American component in the genome of Mennonites (Figure S2). Therefore, this residual non-European ancestry cannot be considered to be real. The results obtained from the *D*-statistics (*f*_*3*_ and *f*_*4*_-tests) corroborate the lack of admixture between Argentinean Mennonite profiles and African and/or Native American variation.

According to historical documentation, the ultimate homeland of Argentinean Mennonites was the Netherlands. Among the set of reference populations used for comparisons, the CEU (from 1000G) is the population set that best represents a Central European population. Not surprisingly, it is the CEU that appears in the results as the population most closely related to the Mennonites. This suggests that, despite the complex routes followed by Mennonites across America, from North to South, and the different populations they were in contact with, they have preserved their most ancestral European ancestry. This is also in good agreement with the increased consanguinity observed in the Argentinean congregation compared to other European groups; which is favored by low effective population sizes, limited gene flow, and endogamic marriages.

Overall, the results obtained in the present study fit well with those obtained in the analysis of Y-chromosome for male Argentinean Mennonites, particularly in two main aspects. First, ancestry in both the autosomes and the uniparental markers is virtually 100% European. Second, the Y-chromosome profiles analyzed in Toscanini *et al*.^9^ already suggested the existence of endogamy in Mennonites.

The results of the present study cannot directly identify the possible admixture that could have occurred in America with non-Mennonites of European ancestry. However, the genomes of Argentinean Mennonites seem to show more affinities with Central Europeans than to e.g. Mediterranean (represented e.g. by Spanish from 1000G) or Atlantic Europeans (represented e.g. by Great Britain from 1000G). This would indirectly suggest limited inbreeding of Mennonites with non-Mennonite American communities. Finally, our study focused on an Argentinean Mennonite congregation, and therefore, extrapolating the present results to other American congregations should be taken with caution. It is important to note however that Argentinean Mennonites have derived very recently from other American Mennonites and therefore, its genomic architecture should resemble the genomic architecture of their most recent American ancestors.

Summarizing, the variation observed in Argentinean Mennonites is coherent with their known style of life and mating rules. These demographic features together set the ground for higher historical consanguinity and could also explain a higher incidence of certain Mendelian diseases in these small communities^15^.

## Methods

### Sampling

A total of 27 saliva samples were recruited on Oragene DNA collection kits (DNAgenotek) from individuals belonging to the Mennonite congregation of ‘La Nueva Esperanza’ (La Pampa, Argentina). Non related males from this DNA collection were previously analyzed for Y-chromosome markers^9^.

We obtained written informed consent for all the donors prior the research, which includes consent for publication of individual data. Rights of participants were safeguarded during the research and their identity was protected. The study conforms with all applicable Spanish normative, namely the Biomedical Research Act (14/2007-3 of July), the Autonomy of the Patient Act (41/2002), Decree SAS/3470/2009 for observational studies and the Data Protection Act (15/1999). The experimental protocol was approved by the Ethical Committee of the Fundación Favaloro (Acta 536) from Buenos Aires, (Argentina).

DNA was extracted from Oragene kits using manufacture protocols.

### Considerations on sample size

The colony of “La Nueva Esperanza” was founded by 120 families coming from México and Bolivia in 1985^2^. Almost two decades after the first colonists’ arrival, the 2005 internal census yielded a population of 1,278 individuals. Taking into consideration the size of the Mennonite founders of “La Nueva Esperanza”, and assuming that parents of these families were unrelated, the 22 individuals analyzed in the present study would represent about 9% of unrelated founders (22 out of 240). This proportion of population analyzed is by far very high when compared to most of molecular studies on human populations. Moreover, the SNP genome-wide approach carried out in the present study is able to capture demographic histories from individual genomes. Recent studies on contemporary populations^16^ or ancient DNA^17^,^21^ were carried out on one or a few individuals, from which demographic past histories could be reconstructed.

### Genotyping

All the samples were genotyped using the Axiom^®^ Genome-Wide Human Origins 1 Arrays at the Centro Nacional de Genotipado (CEGEN) of Santiago de Compostela (Spain). The Affymetrix Genotyping Console™ (GTC) 4.1.2 Software package was used to generate QC metrics and genotype calls following commercial recommendations; including generation of sample dish QC (DQC) values. All the samples yielded DQC values above the default threshold of 0.82. The “AxiomGT1_all” algorithm was used for genotyping this group of high quality samples using all SNPs on the array. A final set of 580,268 SNPs could be used for the analyses. All individuals had less than 0.3% of missing alleles. The quality of the genotyping was very high; thus, only 7, 43, and 602 variants showed 10%, 5%, and 1% of missing data, respectively. From all pairs of individuals related, the one with the highest missing data proportion was eliminated (see below). A total of five donors were finally excluded from the analysis because they showed cryptic relatedness.

### Reference populations

Different SNPs genome repositories from diverse human populations were intersected with the SNP data obtained from the Mennonites; Supplementary Data Table S1. Data from 1000G provides the dataset with the largest SNP overlap with the Mennonites. The data from 1000G was retrieved from the original repository as done in Pardo-Seco *et al*.^22^; these analyses involved 565,777 SNPs. In our figures and text we renamed the Iberian sample, IBD, as IBR in order to avoid confusion with the identity-by-state acronym. In order to explore for the existence of Native American component in the genome of Mennonites, we also used the masked dataset from Reich *et al*.^20^. This dataset, merged with 1000G data and the Mennonites data, yielded a total of 99,111 overlapped SNPs.

### Statistical analysis

A number of analyses were carried out using PLINK (ref. 23). First we computed IBD values from SNP data. We also tested Mennonite SNP data for potential close relationships between individuals. For this, we followed the procedure detailed in Gómez-Carballa *et al*.^24^. IBD values show a few close relationships among the Mennonites sampled. For this reason, we excluded one individual from each parent-offspring and sibling-sibling par, which correspond with IBD values around 0.5 (refs 25 and 26).

MDS was carried out on a matrix of pairwise individual IBS values with the aim of exploring clusters of genetic variation in the population sets analyzed. MDS was performed using the function *cmdscale* (library *stats*) from R (http://www.r-project.org). Average IBS distances (used in Fig. 2C,D) were computed as the average of the matrix of IBS distances computed between individual Mennonites against all the individuals from each reference population.

Admixture in Mennonite genomes was explored using two different approaches. Firstly, we used ADMIXTURE software^27^, which uses a maximum likelihood estimation of individual ancestries from multi-locus SNP data. Secondly, we used PCAdmix 1.0 (ref. 28) to explore local ancestry assignment of ancestry-specific haplotypes across the genome. In order to create input files for PCAdmix, SNP unphased data were imputed and haplotypes were built using Beagle 3.3.2. (ref. 29). This analysis was carried out using reference populations from 1000G representing main continental ancestries: CEU (Europeans), YRI (sub-Saharan Africans), PEL (Native Americans) and CHB (East Asians). We assigned genomic segments to the three main ancestries following a posterior probability threshold of 0.8. Due to the fact that the Peruvian population set from 1000G has a proportion of European ancestry, as an alternative we also run Admixture and PCAdmix using Aymara and Quechua populations from Reich *et al*.^20^ as Native American dataset. These results of all these admixture analysis were in agreement with each other.

The 3-population test or *f*_*3*_-statistics of the form *f*_*3*_(CEU,X_AFR_;MEN) and *f*_*3*_(CEU,X_NAM_;MEN) were computed in order to test for genetic admixture between different African (X_AFR_) and Native American (X_NAM_) population sets with the Mennonites (MEN). A non-negative mean would indicate that the profile of Mennonites cannot be explained by recent admixture with Africans or Native Americans. In order to determine the relationship existing between the Mennonite SNP profiles and the different population sets^20^,^30^ we further computed a 4-population test or *f*_*4*_-statistics (ref. 31). This is a formal test for admixture that measures allele frequency correlations among populations; it provides statistical evidence of admixture and information on the directionality of the gene flow. The *f*_*4*_-statistic was computed using the weighted block jackknife procedure (block size of 5MB) (ref. 32). An outgroup is needed for the computation of *f*_*4*_-statistics. We used an outgroup that is symmetrically related to all modern human population groups, obtained by creating an individual profile possessing the ancestral alleles at all sites. This artificial outgroup ensures that there is no differential gene flow between this outgroup and the population sets used. More details on how this outgroup was built can be found in Pardo-Seco *et al*.^16^.

## Additional Information

**How to cite this article**: Pardo-Seco, J. *et al*. Genomic continuity of Argentinean Mennonites. *Sci. Rep.* **6**, 36392; doi: 10.1038/srep36392 (2016).

**Publisher’s note**: Springer Nature remains neutral with regard to jurisdictional claims in published maps and institutional affiliations.

## Supplementary Material

Supplementary Information
