# Supplementary material for: Genomic continuity of Argentinean Mennonites
Source: Sci Rep. 2016 Nov 8;6:36392. doi: 10.1038/srep36392 (PMC5099698; doi:10.1038/srep36392)
Supplement: Supplementary Information [file srep36392-s1.pdf]

Supplementary Data

Genomic continuity of Argentinean Mennonites

Jacobo Pardo-Seco, Cintial Llull, Gabriela Berardi, Andrea Gómez, Fernando Andreatta, Federico Martinón-Torres, Ulises Toscanini, Antonio Salas

Contents

Figure S1. A) F_{ST} values between 1000G and Mennonites. B) MDS plot of F_{ST} values. C) Dendogram of population samples based on F_{ST} distances.

Figure S2. These figures differ from **Figure 3** in that the reference populations were different, namely, CEU, Aymara, Quechua, CHB and . A) Analysis of admixture of Mennonites using ADMIXTURE. The bar-plot represents individual ancestries in reference populations and Mennonites. It was carried out using the unsupervised clustering algoritm implemented in ADMIXTURE and considering the run with the lowest cross validation value. B) Genomic ancestry mosaic for one Mennonite using PCAdmix C) Admixture proportions of Mennonites using PCAdmix.

Table S1. Population sets used for analyses.

Figure S1.

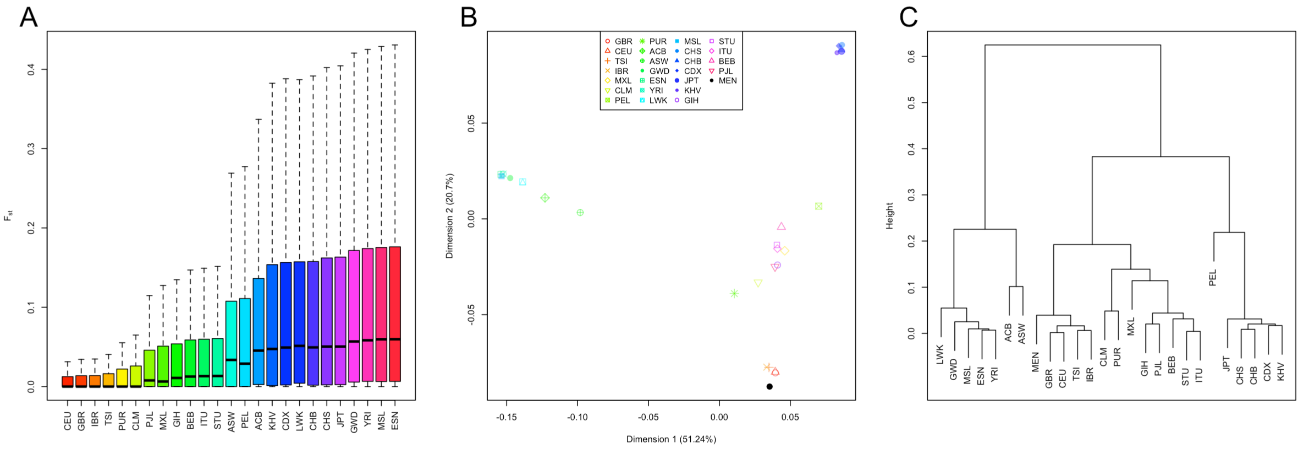
A
B
C
Fst
Dimension 1 (51.24%)
Dimension 2 (20.7%)
Height
CEU GBR IBR TSI PUR CLM PJL MXL GIH BEB ITU STU ASW PEL ACB KHV CDX LWK CHB CHS JPT GWD YRI MSL ESN
GBR CEU TSI IBR MXL CLM PEL PUR ACB ASW GWD ESN YRI LWK MSL CHS CHB CDX JPT KHV STU ITU BEB PJL GIH MEN
LWK GWD MSL ESN YRI ACB ASW MEN GBR CEU TSI IBR CLM PUR MXL GIH PJL BEB STU ITU PEL JPT CHS CHB CDX KHV

Figure S2.

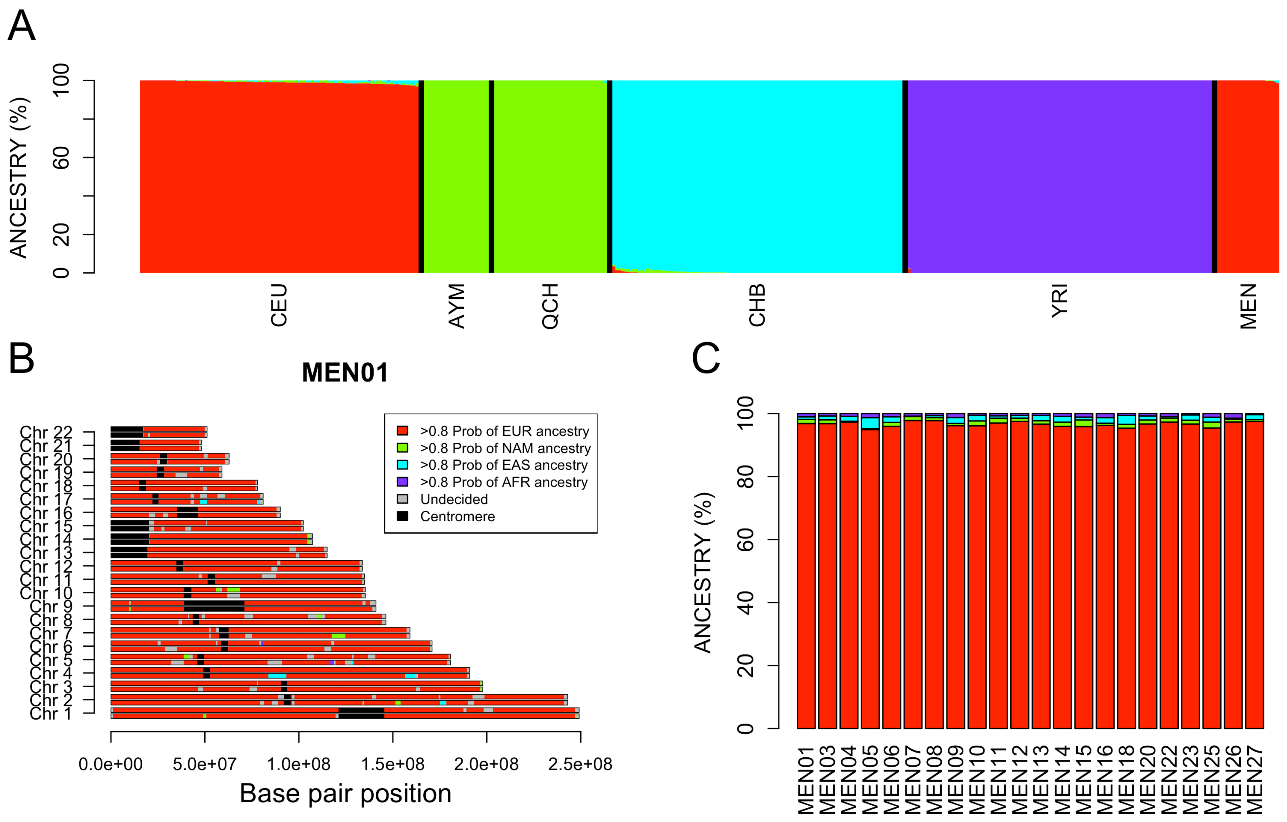
A
ANCESTRY (%)
0
20
60
100
CEU
AYM
QCH
CHB
YRI
MEN
B
MEN01
Chr 22
Chr 21
Chr 20
Chr 19
Chr 18
Chr 17
Chr 16
Chr 15
Chr 14
Chr 13
Chr 12
Chr 11
Chr 10
Chr 9
Chr 8
Chr 7
Chr 6
Chr 5
Chr 4
Chr 3
Chr 2
Chr 1
>0.8 Prob of EUR ancestry
>0.8 Prob of NAM ancestry
>0.8 Prob of EAS ancestry
>0.8 Prob of AFR ancestry
Undecided
Centromere
0.0e+00
5.0e+07
1.0e+08
1.5e+08
2.0e+08
2.5e+08
Base pair position
C
ANCESTRY (%)
0
20
40
60
80
100
MEN01
MEN03
MEN04
MEN05
MEN06
MEN07
MEN08
MEN09
MEN10
MEN11
MEN12
MEN13
MEN14
MEN15
MEN16
MEN18
MEN20
MEN22
MEN23
MEN25
MEN26
MEN27

Table S1. Population sets used for analyses.

Population	Description	Continental origin	Sample Size	Source
ESN	Esan in Nigeria	Africa	99	[1]
GWD	Gambian in Western Divisions in the Gambia	Africa	113	[1]
LWK	Luhya in Webuye, Kenya	Africa	99	[1]
MSL	Mende in Sierra Leone	Africa	85	[1]
YRI	Yoruba in Ibadan, Nigeria	Africa	108	[1]
ACB	African Caribbeans in Barbados	Native American	96	[1]
CDX	Chinese Dai in Xishuangbanna, China	East Asia	93	[1]
CHB	Han Chinese in Bejing, China	East Asia	103	[1]
CHS	Southern Han Chinese	East Asia	105	[1]
JPT	Japanese in Tokyo, Japan	East Asia	104	[1]
KHV	Kinh in Ho Chi Minh City, Vietnam	East Asia	99	[1]
GBR	British in England and Scotland	Europe	91	[1]
IBS [code used in the text: IBR]	Iberian Population in Spain	Europe	107	[1]
TSI	Toscani in Italia	Europe	107	[1]
CLM	Colombians from Medellin, Colombia	Native American	94	[1]
MXL	Mexican Ancestry from Los Angeles USA	Native American	64	[1]
PEL	Peruvians from Lima, Peru	Native American	85	[1]
PUR	Puerto Ricans from Puerto Rico	Native American	104	[1]
ASW	Americans of African Ancestry in SW USA	Africa	61	[1]
CEU	Utah Residents (CEPH) with Northern and Western European Ancestry	Europe	99	[1]
BEB	Bengali from Bangladesh	South Asia	86	[1]
GIH	Gujarati Indian from Houston, Texas	South Asia	103	[1]
ITU	Indian Telugu from the UK	South Asia	102	[1]
PJL	Punjabi from Lahore, Pakistan	South Asia	96	[1]
STU	Sri Lankan Tamil from the UK	South Asia	102	[1]
Aleutian	Eskimo-Aleut, USA	Native American	8	[2]
Aymara	Andean, Bolivia	Native American	23	[2]
Cabecar	Chibchan-Paezan, Costa Rica	Native American	31	[2]
Chilote	Andean, Chile	Native American	8	[2]
Chipewyan	Na-Dene, Canada	Native American	15	[2]
EastGreenland	Eskimo-Aleut, Greenland	Native American	7	[2]
Guahibo	Equatorial-Tucanoan, Paraguay	Native American	6	[2]
Guarani	Equatorial-Tucanoan, Paraguay	Native American	6	[2]
Inga	Andean, Colombia	Native American	9	[2]
Kaqchikel	Northern-Amerind, Guatemala	Native American	13	[2]
Karitiana	Equatorial-Tucanoan, Brazil	Native American	13	[2]
Maya1	Northern-Amerind, Mexico	Native American	37	[2]
Maya2	Northern-Amerind, Mexico	Native American	12	[2]
Mixe	Northern-Amerind, Mexico	Native American	17	[2]
Piapoco	Equatorial-Tucanoan, Colombia	Native American	7	[2]
Pima	Central-Amerind, Mexico	Native American	33	[2]
Quechua	Andean, Bolivia	Native American	40	[2]
Surui	Equatorial-Tucanoan, Brazil	Native American	24	[2]
Tepehuano	Central-Amerind, Mexico	Native American	25	[2]
Ticuna	Equatorial-Tucanoan, Colombia	Native American	6	[2]
Wayuu	Equatorial-Tucanoan, Colombia	Native American	11	[2]
WestGreenland	Eskimo-Aleut, Greenland	Native American	8	[2]
Zapotec1	Central-Amerind, Mexico	Native American	22	[2]
Zapotec2	Central-Amerind, Mexico	Native American	21	[2]
Mennonites		Argentina	27	Present study
**Total sample size**			2834	

Number of SNPs intersected with the different SNP repositories

Datasets	Number of SNPs
Mennonites	580268
Mennonites + 1000G	565777
Mennonites + 1000G + Reich	99111

References

[1] Genomes Project Consortium, Abecasis, et al. (2012) An integrated map of genetic variation from 1,092 human genomes. Nature 491: 56–65.

[2] Reich et al. (2013(Reconstructing native american population history. Nature 488(7411): 370-374
